# Bladder activity of different PSMA PET radioligands and impact of furosemide

**DOI:** 10.21203/rs.3.rs-6537072/v1

**Published:** 2025-05-08

**Authors:** Ismaheel Lawal, Aliza Mushtaq, Samuel Gitau, Khalid Makhdomi, Manali Rupji, Jeffrey Switchenko, Krishna Chaudhary, Ashesh Jani, David Schuster, Charles Marcus

**Affiliations:** Emory University; Emory University; Aga Khan University Hospital; Aga Khan University Hospital; Emory University; Emory University; Emory University; Emory University; Emory University; Memorial Sloan Kettering Cancer Center

**Keywords:** Bladder activity, Furosemide, 18F-PSMA-1007, 68Ga-PSMA-11, 18F-DCFPyL, 18F-rhPSMA-7.3

## Abstract

**Background:**

Prostate-specific membrane antigen (PSMA) radioligands used for PET imaging of prostate cancer (PCa) have variable urinary excretion. The prostate bed region is an important site of disease localization where intense bladder activity may obscure lesion detection. We performed a comparative analysis of the bladder activity of different PSMA radioligands and investigated the impact of furosemide administration on bladder activity reduction.

**Methods:**

This is a retrospective analysis of PSMA PET/CT images of patients with PCa who were imaged with ^68^Ga-PSMA-11 with/without 20 mg furosemide, ^18^F-PSMA-1007, ^18^F-DCFPyL, ^18^F-rhPSMA-7.3 with/without 20 mg furosemide. Exclusion criteria were renal failure (eGFR < 60 mLs/min/m^2^), the use of a urethral catheter or nephrostomy tube in situ, or prior urinary diversion surgery. PSMA PET/CT images were acquired per published procedure standards. We determined and compared bladder volume and bladder activity level using mean and maximum standardized uptake values (SUVmean and SUVmax) between PET scans obtained with the different PSMA radioligands using an ANOVA or Kruskal-Wallis’s test, as appropriate. We also determined the association between the bladder activity level versus bladder volume using Spearman correlation.

**Results:**

210 PSMA PET/CT studies were reviewed, including 50, 20, 20, 28, 42, and 50 completed with ^18^F-PSMA-1007 without furosemide, ^18^F-rhPSMA-7.3 without furosemide, ^18^F-rhPSMA-7.3 with furosemide, ^68^Ga-PSMA-11 without furosemide, ^68^Ga-PSMA-11 with furosemide, and ^18^F-DCFPyL without furosemide, respectively. The median bladder SUVmean (range) without furosemide were: 1.75 (0.4–6.4) [^18^F-PSMA-1007], 10.00 (1.9–140.0) [^18^F-rhPSMA-7.3], 15.92 (2.0–106.0) [^68^Ga-PSMA-11], and 25.7 (7.9–87.6) [^18^F-DCFPyL], (P < 0.001). With 20 mg furosemide co-administered with the radiotracer, there was a significant decline in bladder activity level (median SUVmean of 10.00 (1.9–140.0) to 2.95 (0.8–17.6) for ^18^F-rhPSMA-7.3 and 15.92 (2.0–106.0) to 10.21 (2.6–281.3) for ^68^Ga-PSMA-11 and a significant increase in bladder volume, p < 0.05. There was a significant negative correlation between bladder SUVmax and bladder volume for the entire cohort, p = 0.008, r=−181.

**Conclusion:**

There is variation in the bladder radioactivity between the different PSMA radioligands for PCa PET imaging, with ^18^F-PSMA-1007 demonstrating the lowest, ^68^Ga-PSMA-11 and ^18^F-DCFPyL the highest, and ^18^F-rhPSMA-7.3 intermediate bladder activity level. Administration of 20 mg furosemide produces a significant reduction in bladder activity and an increase in bladder volume. With 20 mg furosemide, bladder activity of ^18^F-rhPSMA-7.3 approaches that of ^18^F-PSMA-1007.

## Introduction

Prostate-specific membrane antigen (PSMA), a transmembrane glycoprotein with a large extracellular domain, is overexpressed in most prostate cancer cells, making it a suitable target for prostate cancer (PCa) imaging and therapy [1]. Several ligands targeting the PSMA extracellular domain have been developed and complexed with diagnostic radionuclides for positron emission tomography (PET) imaging of PCa. The best-characterized diagnostic PSMA radioligands for PET imaging of PCa include ^68^Ga-PSMA-11, ^18^F-DCFPyL, ^18^F-rhPSMA-7.3, and ^18^F-PSMA-1007. The diagnostic performance and superiority of ^68^Ga-PSMA-11, ^18^F-DCFPyL, and ^18^F-rhPSMA-7.3 over conventional imaging in PCa initial staging and recurrence detection have been confirmed in several phase III trials [2–8]. PSMA-targeted imaging is currently the diagnostic imaging of choice in PCa management.

All PSMA radioligands are excreted in the urine to a varying extent. The proximity of the prostate gland to the urinary bladder presents a challenge due to the potential of urinary bladder activity from the excreted radiotracer obscuring the visualization of PCa lesions adjacent to bladder luminal activity. This challenge is particularly important in early PCa recurrence post-prostatectomy, where most PCa recurrence occur as a small lesion in the prostate bed where visualization may be obscured by bladder activity. Detection of local prostate gland/bed recurrence contributes significantly to the overall detection rate of PSMA-targeted PET imaging of PCa at all PSA levels [9, 10]. The closer the lesion is to the urinary bladder with high urine activity, the less likely it is to be detected with PSMA-targeted PET imaging [11].

In phase III trials, PSMA-targeted PET imaging has an excellent lesion detection rate with increasing PSA levels [2, 6, 7]. At PSA below 0.5 ng/mL, detection rates range between 38% and 64% [2, 6, 7], indicating that the approved PSMA radioligands will fail to localize the site of PCa recurrence in 1/3rd to 2/3rd of patients with PSA below 0.5 ng/mL. Therefore, there is a clinical need for strategies to improve PCa lesion detection, especially at low PSA. Also, it is important to understand the differences in biokinetics of the different PSMA radioligands viz-a-viz their urinary excretion rate and explore avenues to utilize this understanding in interpreting PSMA PET findings and selecting the appropriate PSMA radioligand for a given clinical scenario.

Due to the critical impact of bladder activity level on the visualization of PCa lesions in the prostate bed, several avenues aimed at reducing urinary activity level at the time of PET imaging have been explored, including furosemide administration, early imaging, delayed imaging, dynamic imaging, and dual-timepoint imaging [12–14]. In our centers, we have had real-world experience with severe PSMA radiotracers and strategies to ameliorate bladder activity in both clinical and research scenarios. In this retrospective study, our aim is to compare bladder activity for these radiotracers and mitigation strategies.

## Methods

### Patients

This is a retrospective analysis of PSMA PET/CT imaging performed at Emory University in patients who received ^68^Ga-PSMA-11 with 20 mg intravenous furosemide for PET/CT imaging in the EMPIRE-2 trial (NCT03762759), ^68^Ga-PSMA-11 without furosemide and ^18^F-DCFPyL without furosemide as standard of care PET/CT imaging of prostate cancer, and ^18^F-rhPSMA-7.3 with and without furosemide in a phase II trial (NCT05779943), which is reported separately for an intrapatient comparison for ^18^F-rhPSMA-7.3 alone. The clinical ^68^Ga-PSMA-11 without furosemide and ^18^F-DCFPyL without furosemide scans included in this study were randomly selected from cohorts of PCa patients who received standard-of-care PSMA PET imaging until August 2023. This study also retrospectively analyzed ^18^F-PSMA-1007 PET/CT images of patients with prostate cancer imaged at the Aga Khan University Hospital in Nairobi, Kenya between June and December 2023. Patients were included if they were 18 years and above and had a technically adequate PSMA radioligand PET/CT for initial staging or biochemical recurrence of prostate cancer. Exclusion criteria included PSMA radioligand PET/CT imaging for non-prostate cancers, patients with a urethral catheter or percutaneous nephrostomy tube in place, patients with renal failure defined as estimated glomerular filtration rate (eGFR) of < 60 ml/mins/m^2^, and patients with prior urinary diversion surgery. The institutional review board of the respective institutions approved this study and granted an informed consent waiver due to the retrospective nature of this work.

### PSMA radioligand PET/CT imaging and image analysis

PSMA radioligand PET/CT imaging was performed according to published guidelines/procedure standards [15]. Briefly, no special patient preparation was observed. All patients received intravenous radiotracer administration followed by PET/CT imaging after an approximate 60-minute uptake period. For the diuretic-enhanced ^68^Ga-PSMA-11 and ^18^F-rhPSMA-7.3 PET/CT, 20 mg intravenous furosemide was co-administered with the radiotracer. The patients were encouraged to void during the uptake period but asked to refrain from voiding 15 minutes before the commencement of PET/CT imaging to allow for adequate bladder distention.

Imaging was performed on one of GE Discovery 690 (GE Healthcare, Milwaukee, WI), Siemens Biograph mCT, Siemens Vision 600 (Siemens Medical Solution USA, Inc.), or UMI 550 (United Imaging Healthcare Co., Ltd, Houston, TX) within Emory Healthcare and GE Discovery MI PET/CT system (GE Healthcare, Milwaukee, WI) at Aga Khan University Hospital in Nairobi. Following CT imaging, thigh-to-vertex PET imaging was acquired in 3D mode at 3 minutes per bed position. The PET data was corrected for randoms, scatter, dead time, and attenuation. Image reconstruction was done with the OSEM iterative reconstruction algorithm.

For each PSMA PET/CT study, a volume of interest (VOI) was drawn within the bladder to obtain the mean and maximum standardized uptake values (SUVmean and SUVmax) of bladder radioactivity level. Bladder volume was also determined.

### Statistical analysis

Descriptive statistics were generated for all patient characteristics. Frequency and percentage were reported for categorical variables. Mean (standard deviation, SD) and median (range or interquartile range, IQR) were reported for numeric variables. The clinical characteristics of patients who received the different PSMA radioligand for PET imaging were compared using an ANOVA or Kruskal-Wallis test, as appropriate. The correlation between bladder SUVmax and the bladder volume was compared using Pearson’s correlation test or Spearman’s correlation test, as appropriate for the entire cohort and separately for each PSMA radioligand group. Similarly, the correlation between SUVmean and the variables was also tested. Variables that did not meet the Gaussian assumption were log-transformed. Bladder volume, SUVmax, SUVmean were compared between the ^18^F-rhPSMA-7.3 with furosemide and ^18^F-rhPSMA-7.3 without furosemide using a paired t-test. Similarly, ^68^Ga-PSMA-11 with furosemide and ^68^Ga-PSMA-11 without furosemide cohorts were compared for the sample variables using an ANOVA or Kruskal-Wallis’s test, as appropriate.

Statistical analysis was performed using SAS 9.4 (SAS Institute Inc., Cary, NC), and statistical significance was assessed at the 0.05 level.

## Results

**Figure 1:**
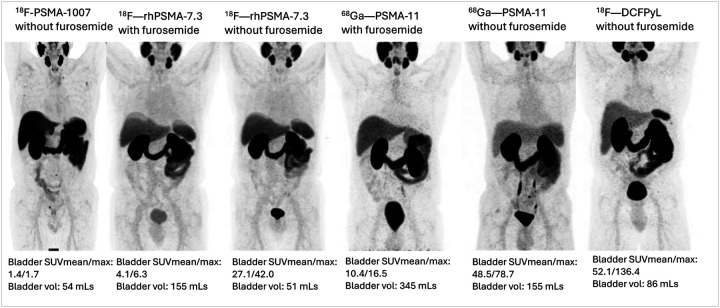
MIP images with similar windowing level showing relative bladder activity of different PSMA radioligands on PET imaging acquired with or without furosemide administration.

A total of 210 studies were included in this analysis, including 50, 20, 20, 28, 42, and 50 PSMA PET/CT studies done with^18^F-PSMA-1007 without furosemide, ^18^F-rhPSMA-7.3 without furosemide, ^18^F-rhPSMA-with furosemide, ^68^Ga-PSMA-11 without furosemide, ^68^Ga-PSMA-11 with furosemide, and ^18^F-DCFPyL without furosemide, respectively. There was a significant difference in bladder activity measured by SUVmean and SUVmax across the six PSMA PET/CT subgroups considered, p < 0.001 (Table 1, Fig. 1). [range: 1.9–140.0] and 10.21 [range: 2.6–281.3], respectively) and median SUVmax (13.35 [rang: 3.9–165.4] and 17.23 [range: 4.7–514.7], respectively). Bladder volume was also significantly different across PSMA PET subgroups, with the highest bladder volume recorded for PSMA PET/CT done with furosemide administration (Table 1). Bladder activity was significantly lower, and bladder volume was significantly higher in ^68^Ga-PSMA-11 with furosemide and ^18^F-rhPSMA-7.3 with furosemide compared with ^68^Ga-PSMA-11 without furosemide and ^18^F-rhPSMA-7.3 without furosemide (supplementary tables 1 and 2).

### Correlation between bladder activity versus bladder volume

Considering all PSMA studies, regardless of the type of PSMA radioligand, there was a significant negative correlation between bladder SUVmax and bladder volume and a trend towards significance between bladder SUVmean and bladder volume (Table 2). At all subgroup levels, there was either a significant negative correlation or trend toward statistical significance between bladder activity (measured by SUVmax and SUVmean) and bladder volume for ^18^F-PSMA-1007 without furosemide, ^18^F-DCFPyL without furosemide, ^18^F-rhPSMA-7.3 with furosemide, and ^18^F-rhPSMA-7.3 without furosemide. There was no significant association between bladder activity and bladder volume for ^68^Ga-PSMA-11 either with or without furosemide (Table 2).

## Discussion

We performed a retrospective analysis of bladder activity on PET imaging obtained with four PSMA radioligands utilized in our clinical and research programs; ^68^Ga-PSMA-11, ^18^F-DCFPyL, ^18^F-PSMA-1007, and ^18^F-rhPSMA-7.3. We also have cohorts imaged with ^68^Ga-PSMA-11 and ^18^F-rhPSMA-7.3 who were imaged with and without use of diuretic.

We found that ^18^F-PSMA-1007 demonstrated the lowest bladder activity with a median bladder SUVmean of 1.75 [range: 0.4–6.4] compared to 10.00 (1.9–140), 15.92 (2.0–106.0), and 25.7 (7.9–87.6) for ^18^F-rhPSMA-7.3, ^68^Ga-PSMA-11, and ^18^F-DCFPyL, respectively. Twenty mg furosemide co-administered with the radiotracer led to a 71% and 36% reduction in bladder activity of ^18^F-rhPSMA-7.3 and ^68^Ga-PSMA-11 with resultant median SUVmean of 2.95 and 10.21, respectively. As expected, furosemide administration led to increased bladder volume, and the bladder volume, in turn, demonstrated an inverse relationship with bladder activity.

Despite their high diagnostic performance for staging and re-staging of PCa, the urinary excretion of most PSMA radioligands presents a challenge, potentially limiting the localization of small lesions with subtle radiotracer avidity in the prostate bed as detection of recurrent disease on PET is adversely influenced by proximity to the bladder [11]. With the availability of multiple PSMA radioligands for routine clinical use, it is important to understand the nuances in their biodistribution and how these may affect lesion detection. Our results are therefore important in that we present data demonstrating the differences in bladder activity levels of different PSMA radioligand and the influence of furosemide administration.

The values we report for ^18^F-PSMA-1007 with a median bladder SUVmean of 1.75 (range = 0.4–6.4), was similar to a median SUVmean of 3.08 (IQR = 1.66–4.74) at 60 minutes post-injection without diuresis reported by Rahbar et al. [16]. ^18^F-PSMA-1007 has an inherent low rate of urinary excretion among common PSMA radioligands [17]. In the biodistribution study of ^18^F-PSMA-1007 by Giesel et al., 1.2% of the administered radiotracer was excreted into the urinary bladder within the first 2 hours post-tracer administration [18]. This is lower than the 2-hour bladder excretory rates of 7.2% for ^18^F-rhPSMA-7.3 and 11% for ^18^F-DCFPyL and ^68^Ga-PSMA-11, respectively [19–21]. However, ^18^F-PSMA-1007 is not FDA-approved and is unavailable for routine clinical use in the United States.

^18^F-rhPSMA-7.3 has the next lowest bladder activity with a median SUVmean of 10 (range = 1.9–140) but which may be reduced to a median SUVmean of 2.95 (range = 0.8–17.6) by the use of 20 mg furosemide at the time of radiotracer administration. With this diuretic strategy, the FDA-approved ^18^F-rhPSMA-7.3 bladder activity approaches that of ^18^F-PSMA-1007. This median SUVmean of the non-diuretic cohort in our study of 10.00 was similar to median SUVmean 12.5 (0.7–887) in the LIGHTHOUSE and SPOTLIGHT trial cohorts [22].

^68^Ga-PSMA-11 without diuresis had a median SUV mean of 15.92 and was reduced with furosemide to 10.21 in our series. In the study by Uprimny et al., a group who have done extensive work with forced diuresis with ^68^Ga-PSMA-11, 20 mg intravenous furosemide reduced bladder activity from a median SUVmean of 41.9 to 5.3 [23]. In the study by Uprimmy et al., the median SUVmean of ^68^Ga-PSMA-11 without furosemide was higher, and the median SUVmean of ^68^Ga-PSMA-11 with furosemide was lower than in our study. These differences may be due to variance in patient population including renal function and chronic diuretic use which was not controlled for in our analysis. Most importantly the lower post-diuretic activity in the study by Uprimny is likely due to more robust forced diuresis utilizing 500 ml IV saline which was also reported in a separate manuscript [24] to result in 40% urinary urgency as compared to our ^68^Ga-PSMA-11 diuretic protocol without IV saline which was overall well tolerated.

^18^F-DCFPyL had the highest median SUVmean of 25.7 on our study. We could not find published data on median SUVmean on this radiotracer, but median SUVmax was reported to be 61.7 in the study by Donswijk et al and 79.32 by Giesel et al which is similar to the median SUVmax in our series of 51.42 [17, 25]. Ferreira and coworkers reported a mean SUVpeak of 57.3, which is also not dissimilar to our findings of mean SUVmax of 66.07, as SUV peak would have slightly lower intensity [26]. Yet, the higher the baseline bladder activity, the more difficult it may be to reduce the activity to less interfering levels with diuretic as reported by numerous authors [14, 25]. It has been our experience that diuresis is not widely utilized with ^18^F-DCFPyL including in our center, and therefore, we do not have clinical data on its use with furosemide. Because ^68^Ga-PSMA-11 baseline activity is significantly greater than that of ^18^F-rhPSMA-7.3, there was only a 36% reduction with use of furosemide coadministered with ^68^Ga-PSMA-11 versus a 71% reduction with use of a diuretic workflow with ^18^F-rhPSMA-7.3.

We hypothesized that bladder distention may influence bladder activity levels due to relative dilution of radiotracer. Considering all 210 included studies, bladder volume showed a negative association with bladder activity, regardless of furosemide use. The negative association between bladder activity and bladder volume was most pronounced for ^18^F-rhPSMA-7.3 with/without furosemide and, to a lesser extent, for ^18^F-DCFPyL and ^18^F-PSMA-1007 (Table 2). We found no significant association between the bladder activity of ^68^Ga-PSMA-11 administered with/without furosemide and bladder volume. A higher bladder volume of dilute urine (less activity) not only reduces the obscuring effect of bladder activity but also allows the urinary bladder to lift away from the prostate bed, allowing better visualization of the prostate bed or intraprostatic lesions. As shown in the study of Freitag et al., lesions closest to the urinary bladder stand the highest risk of being missed on PSMA PET imaging [11]. A full bladder with a lower radioactivity level is also desirable as it mimics the bladder condition during radiotherapy planning, allowing for more accurate PSMA PET and CT simulation image fusion for radiotherapy planning.

Our study has many strengths, including evaluating multiple PSMA radioligands in real world clinical and clinical trial use for PCa staging and restaging. We also investigated the association between bladder volume and bladder activity of these PSMA. Limitations of our study include a relatively heterogenous cohort of clinical and research studies. Three of the radiotracers, ^18^F-rhPSMA-7.3, ^18^F-DCFPyL, and ^68^Ga-PSMA-11 were utilized at multiple hospitals at one university system, while ^18^F-PSMA-1007 data was from only one hospital at a different university. Diuresis was only utilized with ^68^Ga-PSMA-11 and rhPSMA-7.3 as diuresis was not necessary with ^18^F-PSMA-1007 and considered futile with ^18^F-DCFPyL. Our analysis did not investigate the impact of bladder activity level on lesion detection as this has been reported elsewhere [27, 28], suggesting a lower bladder activity level improves lesion detection in the prostate bed region in a subset of patients.

## Conclusion

There is variation in the bladder activity between the different PSMA radioligands. ^18^F-PSMA-1007 has the lowest baseline bladder activity level, intermediate for ^18^F-rhPSMA-7.3, while bladder activity is highest for ^68^Ga-PSMA-11 and ^18^F-DCFPyL. Administration of 20 mg furosemide produces a higher reduction in bladder activity for ^18^F-rhPSMA-7.3 versus ^68^Ga-PSMA-11. In fact, coadministered with 20 mg furosemide, bladder activity of ^18^F-rhPSMA-7.3 approaches that of baseline ^18^F-PSMA-1007. Finally, as an independent factor, there is an inverse relationship between bladder volume and bladder activity for most PSMA radioligands such that bladder activity reduces with increasing bladder volume.

## References

[1] Silver DA, Pellicer I, Fair WR, Prostate-specific membrane antigen expression in normal and malignant human tissues. Clin Cancer Res 1997;3:81–85. 9815541

[2] Fendler WP, Calais J, Eiber M, Assessment of 68Ga-PSMA-11 PET Accuracy in Localizing Recurrent Prostate Cancer: A Prospective Single-Arm Clinical Trial. JAMA Oncol 2019;5:856–863. 30920593 10.1001/jamaoncol.2019.0096 PMC6567829

[3] Hofman MS, Lawrentschuk N, Francis RJ, Prostate-specific membrane antigen PET-CT in patients with high-risk prostate cancer before curative-intent surgery or radiotherapy (proPSMA): a prospective, randomised, multicentre study. Lancet 2020;395:1208–1216. 32209449 10.1016/S0140-6736(20)30314-7

[4] Hope TA, Eiber M, Armstrong WR, Diagnostic Accuracy of 68Ga-PSMA-11 PET for Pelvic Nodal Metastasis Detection Prior to Radical Prostatectomy and Pelvic Lymph Node Dissection: A Multicenter Prospective Phase 3 Imaging Trial. JAMA Oncol 2021;7:1635–1642. 34529005 10.1001/jamaoncol.2021.3771 PMC8446902

[5] Pienta KJ, Gorin MA, Rowe SP, A Phase 2/3 Prospective Multicenter Study of the Diagnostic Accuracy of Prostate Specific Membrane Antigen PET/CT with 18F-DCFPyL in Prostate Cancer Patients (OSPREY). J Urol 2021;206:52–61. 33634707 10.1097/JU.0000000000001698 PMC8556578

[6] Morris MJ, Rowe SP, Gorin MA, Diagnostic Performance of ^18^F-DCFPyL-PET/CT in Men with Biochemically Recurrent Prostate Cancer: Results from the CONDOR Phase III, Multicenter Study. Clin Cancer Res 2021;27:3674–3682. 33622706 10.1158/1078-0432.CCR-20-4573 PMC8382991

[7] Jani AB, Ravizzini GC, Gartrell BA, Diagnostic Performance and Safety of ^18^F-rhPSMA-7.3 Positron Emission Tomography in Men With Suspected Prostate Cancer Recurrence: Results From a Phase 3, Prospective, Multicenter Study (SPOTLIGHT). J Urol 2023;210:299–311. 10.1097/JU.0000000000003493 PMC12721651 37126069

[8] Surasi DS, Eiber M, Maurer T, Diagnostic Performance and Safety of Positron Emission Tomography with ^18^F-rhPSMA-7.3 in Patients with Newly Diagnosed Unfavourable Intermediate- to Very-high-risk Prostate Cancer: Results from a Phase 3, Prospective, Multicentre Study (LIGHTHOUSE). Eur Urol 2023;84:361–370. 37414702 10.1016/j.eururo.2023.06.018

[9] Rauscher I, Karimzadeh A, Schiller K, Detection efficacy of ^18^F-rhPSMA-7.3 PET/CT and impact on patient management in patients with biochemical recurrence of prostate cancer after radical prostatectomy and prior to potential salvage treatment. J Nucl Med 2021;62:1719–1726. 33712531 10.2967/jnumed.120.260091 PMC8612184

[10] Lawal IO, Lengana T, Popoola GO, Pattern of Prostate Cancer Recurrence Assessed by ^68^Ga-PSMA-11 PET/CT in Men Treated with Primary Local Therapy. J Clin Med 2021;10:3883. 34501331 10.3390/jcm10173883 PMC8432125

[11] Freitag MT, Radtke JP, Afshar-Oromieh A, Local recurrence of prostate cancer after radical prostatectomy is at risk to be missed in ^68^Ga-PSMA-11-PET of PET/CT and PET/MRI: comparison with mpMRI integrated in simultaneous PET/MRI. Eur J Nucl Med Mol Imaging 2017;44:776–787. 27988802 10.1007/s00259-016-3594-z

[12] Alberts I, Niklas-Hünermund J, Sachpekidis C, Combination of Forced Diuresis with Additional Late Imaging in ^68^Ga-PSMA-11 PET/CT: Effects on Lesion Visibility and Radiotracer Uptake. J Nucl Med 2021;62:1252–1257. 33547214 10.2967/jnumed.120.257741

[13] Bayerschmidt S, Uprimny C, Kroiss AS, Comparison of Early Imaging and Imaging 60 min Post-Injection after Forced Diuresis with Furosemide in the Assessment of Local Recurrence in Prostate Cancer Patients with Biochemical Recurrence Referred for 68Ga-PSMA-11 PET/CT. Diagnostics (Basel) 2021;11:1191. 34208989 10.3390/diagnostics11071191 PMC8304119

[14] Wondergem M, van der Zant FM, Rafimanesh-Sadr L, Effect of forced diuresis during 18F-DCFPyL PET/CT in patients with prostate cancer: activity in ureters, kidneys and bladder and occurrence of halo artefacts around kidneys and bladder. Nucl Med Commun 2019;40:652–656. 30855543 10.1097/MNM.0000000000001007

[15] Fendler WP, Eiber M, Beheshti M, PSMA PET/CT: joint EANM procedure guideline/SNMMI procedure standard for prostate cancer imaging 2.0. Eur J Nucl Med Mol Imaging 2023;50:1466–1486. 36604326 10.1007/s00259-022-06089-w PMC10027805

[16] Rahbar K, Afshar-Oromieh A, Bögemann M, ^18^F-PSMA-1007 PET/CT at 60 and 120 minutes in patients with prostate cancer: biodistribution, tumour detection and activity kinetics. Eur J Nucl Med Mol Imaging 2018;45:1329–1334. 29541812 10.1007/s00259-018-3989-0

[17] Giesel FL, Will L, Lawal I, Intraindividual Comparison of ^18^F-PSMA-1007 and ^18^F-DCFPyL PET/CT in the Prospective Evaluation of Patients with Newly Diagnosed Prostate Carcinoma: A Pilot Study. J Nucl Med 2018;59:1076–1080. 29269569 10.2967/jnumed.117.204669

[18] Giesel FL, Hadaschik B, Cardinale J, F-18 labelled PSMA-1007: biodistribution, radiation dosimetry and histopathological validation of tumor lesions in prostate cancer patients. Eur J Nucl Med Mol Imaging 2017;44:678–688. 27889802 10.1007/s00259-016-3573-4 PMC5323462

[19] Szabo Z, Mena E, Rowe SP, Initial Evaluation of [(18)F]DCFPyL for Prostate-Specific Membrane Antigen (PSMA)-Targeted PET Imaging of Prostate Cancer. Mol Imaging Biol 2015;17:565–574. 25896814 10.1007/s11307-015-0850-8 PMC4531836

[20] Pfob CH, Ziegler S, Graner FP, Biodistribution and radiation dosimetry of (68)Ga-PSMA HBED CC-a PSMA specific probe for PET imaging of prostate cancer. Eur J Nucl Med Mol Imaging 2016;43:1962–1970. 27207281 10.1007/s00259-016-3424-3

[21] Tolvanen T, Kalliokoski K, Malaspina S, Safety, Biodistribution, and Radiation Dosimetry of ^18^F-rhPSMA-7.3 in Healthy Adult Volunteers. J Nucl Med 2021;62:679–684. 33067338 10.2967/jnumed.120.252114 PMC8844263

[22] Kuo PH, Hermsen R, Penny R, Quantitative and Qualitative Assessment of Urinary Activity of ^18^F-Flotufolastat-PET/CT in Patients with Prostate Cancer: a Post Hoc Analysis of the LIGHTHOUSE and SPOTLIGHT Studies. Mol Imaging Biol 2024;26:53–60. 37932609 10.1007/s11307-023-01867-w PMC10827967

[23] Uprimny C, Bayerschmidt S, Kroiss AS, Early Injection of Furosemide Increases Detection Rate of Local Recurrence in Prostate Cancer Patients with Biochemical Recurrence Referred for ^68^Ga-PSMA-11 PET/CT. J Nucl Med 2021;62:1550–1557. 33712533 10.2967/jnumed.120.261866 PMC8612314

[24] Uprimny C, Bayerschmidt S, Kroiss AS, Impact of forced diuresis with furosemide and hydration on the halo artefact and intensity of tracer accumulation in the urinary bladder and kidneys on [^68^Ga]Ga-PSMA-11-PET/CT in the evaluation of prostate cancer patients. Eur J Nucl Med Mol Imaging 2021;48:123–133. 32385647 10.1007/s00259-020-04846-3

[25] Donswijk ML, Wondergem M, de Wit-van der Veen L, Effects of furosemide and tracer selection on urinary activity and peri-bladder artefacts in PSMA PET/CT: a single-centre retrospective study. EJNMMI Res 2022;12:42. 35895129 10.1186/s13550-022-00913-y PMC9329505

[26] Ferreira G, Iravani A, Hofman MS, Intra-individual comparison of ^68^Ga-PSMA-11 and ^18^F-DCFPyL normal-organ biodistribution. Cancer Imaging 2019;19:23. 31092293 10.1186/s40644-019-0211-y PMC6521415

[27] Lawal IO, Mushtaq A, Jani AB, Rupji M, Diuresis During ^18^F-Flotufolastat (rhPSMA-7.3) PET/CT Improves Recurrence Detection After Prostatectomy: A Prospective Phase II Trial. J Nucl Med 2025;66:230–237. 39848765 10.2967/jnumed.124.268574 PMC13671398

[28] Chen Q, Dong L, Xu L, Comparison of clinical performance between late and standard totalbody [^68^Ga]-GaPSMA-11 in biochemical recurrent prostate cancer. Eur J Nucl Med Mol Imaging 2025;52:1249–1256. 39540904 10.1007/s00259-024-06980-8

